# The Role of the 3′UTR Region in the Regulation of the *ACVR1/Alk-2* Gene Expression

**DOI:** 10.1371/journal.pone.0050958

**Published:** 2012-12-05

**Authors:** Marzia Mura, Serena Cappato, Francesca Giacopelli, Roberto Ravazzolo, Renata Bocciardi

**Affiliations:** 1 Laboratory of Molecular Genetics, G. Gaslini Institute, Genova, Italy; 2 Department of Neurosciences, Rehabilitation, Ophthalmology, Genetics, Maternal and Child Health (DINOGMI), University of Genova and CEBR, Genova, Italy; Maastricht University, The Netherlands

## Abstract

**Background:**

The *ACVR1/Alk-2* gene, encoding a BMP type I receptor, is mutated in Fibrodysplasia Ossificans Progressiva, a severe form of heterotopic ossification. Regulation of *ACVR1/Alk-2* expression, still poorly understood, is likely to be controlled by transcriptional and post-transcriptional mechanisms. In our work, we focused on the functional role of the 3′UTR region of the gene and on microRNAs as possible modulators of the *ACVR1/Alk-2* expression.

**Results:**

The *ACVR1/Alk-2* 3′UTR region consists of a 1.1 kb sequence harboring several putative, well-conserved binding sites for miRNAs in its proximal half, and AU-rich elements in the distal one, as assessed by bioinformatic analysis. The functional role of this region was tested in presence of transcription inhibitors and in transfection experiments in different cell lines, with a *ACVR1/Alk-2*-3′UTR reporter construct. By this transfection-based approach, we have also verified that three microRNAs, among those predicted to target *ACVR1/Alk-2* gene by *in silico* analysis, can bind its 3′UTR sequence thereby modulating its expression.

**Conclusion:**

In this work we demonstrated that the *ACVR1/Alk-2* transcript is unstable in presence of inhibitors of transcription. Functional analysis of the 3′UTR region by Luciferase reporter assays showed that it plays an inhibitory role on *ACVR1/Alk-2* gene expression. Moreover, we found that specific miRNAs are involved in modulating *ACVR1/Alk-2* gene expression as suggested by binding sites prediction in its 3′UTR sequence. In particular, we found that mir148b and mir365 were able to down-regulate *ACVR1/Alk-2* expression, whereas mir26a showed a positive effect on its mRNA. Our data contribute to elucidate some of the mechanisms intervening in the modulation of *ACVR1/Alk-2* expression. Considering that no specific and effective treatment of FOP is available, clarifying the basic mechanisms of the *ACVR1/Alk-2* gene biology may provide means to develop innovative therapeutics approaches.

## Introduction

A large class of small noncoding RNAs, known as microRNAs (miRs), functions as regulators of a broad range of cellular processes by modulating gene expression. The active molecule consists of short single-stranded RNA of about 22 nucleotides in length processed from longer (80–85 nt) precursor. The miRBASE database (release 17, April 2011) includes 16,772 entries, 1,424 of which are human miRs. Computer prediction of binding sites indicates that the majority of human protein-coding genes are regulated by miRs, that a single miR has the potential to regulate many mRNA transcripts and a single mRNA can serve as a target of multiple miRs. Their biological function is typically based on the ability to control mRNA accumulation and/or protein synthesis through specific interactions with cis-acting elements in the 3′UTRs of target genes. (see [1] for a comprehensive review).

Bone remodeling, that is bone reabsorption and formation that takes place along the entire lifetime, is a dynamic and physiological process in which several important signaling pathways and target genes play fundamental roles. One of the most important pathways is related to Bone Morphogenetic Protenis (BMPs) that act through the interplay between BMP ligands, BMP receptors and downstream intracellular signaling molecules. SMAD-dependent and non SMAD pathways act downstream of BMP receptors [2]. Both defective and hyperfunctioning BMP signaling can be observed as cause of abnormal clinical phenotypes in humans.

Signaling abnormalities may depend on several components of the BMP pathways, among which receptors represent key molecules being the first indispensable step. Given the wide variety of biological processes in which, besides bone remodeling, BMPs play important roles, it is conceivable that BMP receptors are finely regulated to guarantee a correct balance between hypo- and hyper- expression [3], [4].

The most striking and severe clinical manifestation of hyperfunctioning BMP signaling is Fibrodysplasia Ossificans Progressiva (FOP), caused by mutation in the *ACVR1/Alk-2* gene that encodes the ACVR1/ALK-2 type I receptor [5], [6]. Experimental evidences support the idea that the effect of mutation in the *ACVR1/Alk-2* gene is activation of the SMAD-dependent signaling pathway.

The identification and characterization of the mechanisms intervening in the regulation of *ACVR1/Alk-2* gene expression are complementary to studies aimed at identifying and characterizing the alteration of molecular and cellular mechanisms underlying heterotopic ossification process typical of FOP. Skeletal manifestations in this disease are the final result of a variety of biological processes that involve inflammatory/immune mechanisms and stimulation of multipotent progenitors to undertake an endochondral osteogenic process. This makes difficult to find a single cellular model that recapitulates the multistep and multicellular pathogenic mechanism, but requires to verify that, at least as an initial approach, some of the regulatory mechanisms are common to different cell types or there are significant differences.

Mechanisms of *ACVR1/Alk-2* expression regulation have not been studied in deep up to now, therefore we have started to analyze the 3′UTR region to verify the possibility of post-transcriptional regulation by using cell lines of different origin and describe here the regulatory role of some miRs acting at the level of this region.

## Results

*ACVR1/Alk-2* gene is widely expressed in different tissues and cell lines and, like other receptors of important and critical signaling pathways, is likely to be targeted by different regulatory mechanisms of its gene expression.

**Figure 1:**
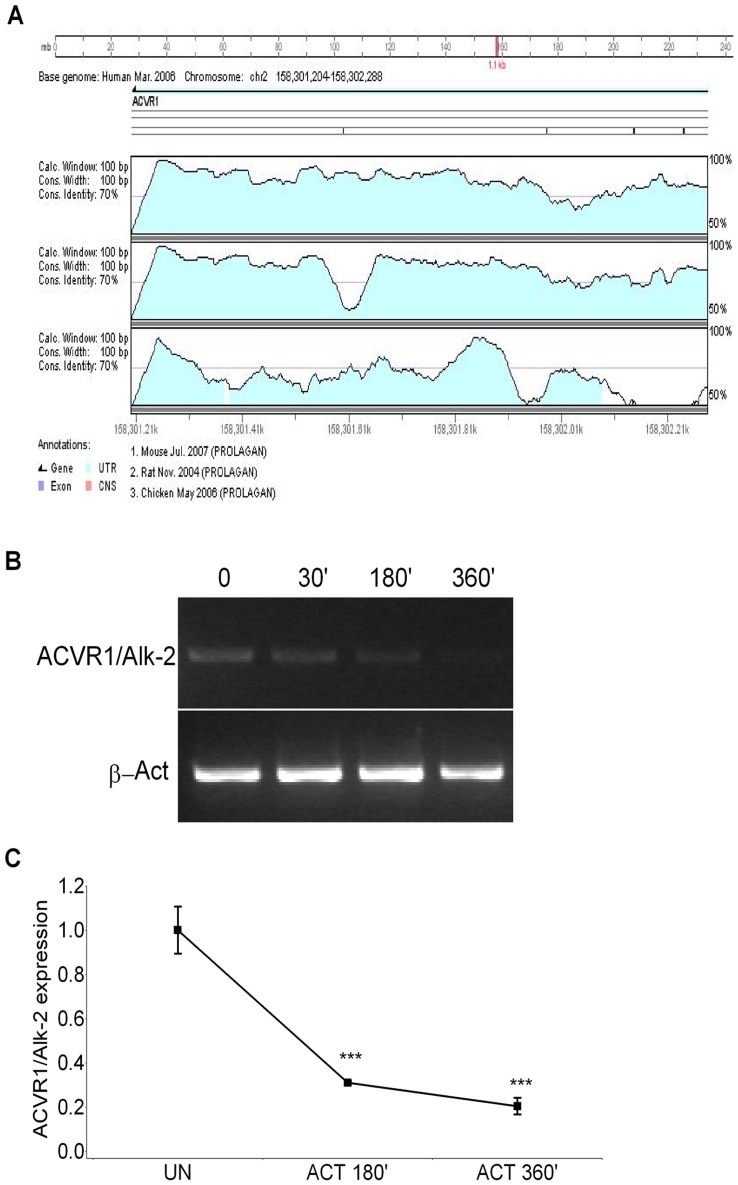
*ACVR1/Alk-2* mRNA is well conserved among species and is unstable in presence of ActD. A) Output of the comparative genomic analysis obtained with a dedicated software available at the Vista Genome Browser (http://genome.lbl.gov/vista/index.shtml, tools for comparative analysis*). Non coding Conserved sequences corresponding to the *ACVR1/Alk-2* 3′UTR are shown as light blue peaks, and are shown relative to their position on human chr2 (March 2006, release) compared to the mouse (first track) rat (second track) and chicken (third track) genomes, respectively, the percentage of identity is indicated on the vertical axis. (*Frazer KA, Pachter L, Poliakov A, Rubin EM, Dubchak I. VISTA: computational tools for comparative genomics. Nucleic Acids Res. 2004 Jul 1;32 (Web Server issue):W273-9). B) and C) C2C12 cells were treated with the ActD transcription inhibitor with the doses and for the time points indicated. *ACVR1/Alk-2* mRNA was measured by semi-quantitative (B) and quantitative RT-PCR (RT-qPCR, **C**). Two-tailed student′s t-test was performed. Significant differences in comparison to untreated cells are given as: *p*<0.05*; *p*<0.01**; or *p*<0.001***.

The 3′UTR region of the *ACVR1-Alk-2* gene consists of a 1.1 kb sequence highly conserved among species, in particular the percentage of identity between human and mouse is around 85% (Fig. 1A and Fig. S1). Extensive bioinformatic analysis of this region suggested the presence of sequences or conserved structural motifs with a potential functional role in post-transcriptional regulation of *ACVR1/Alk-2* expression. The *ACVR1/Alk-2* 3′UTR is defined by two putative functional modules characterized by a proximal region containing several binding sites for miR and a more distal sequence harboring AU-rich Elements (ARE) represented by AUUUA pentamers. In particular for miRs, we applied different miR target prediction tools (see Materials and Methods section) to search for *ACVR1/Alk-2*-targeting miRs by which several miRs were predicted to target the *ACVR1/Alk-2* 3′UTR (not shown).

**Figure 2:**
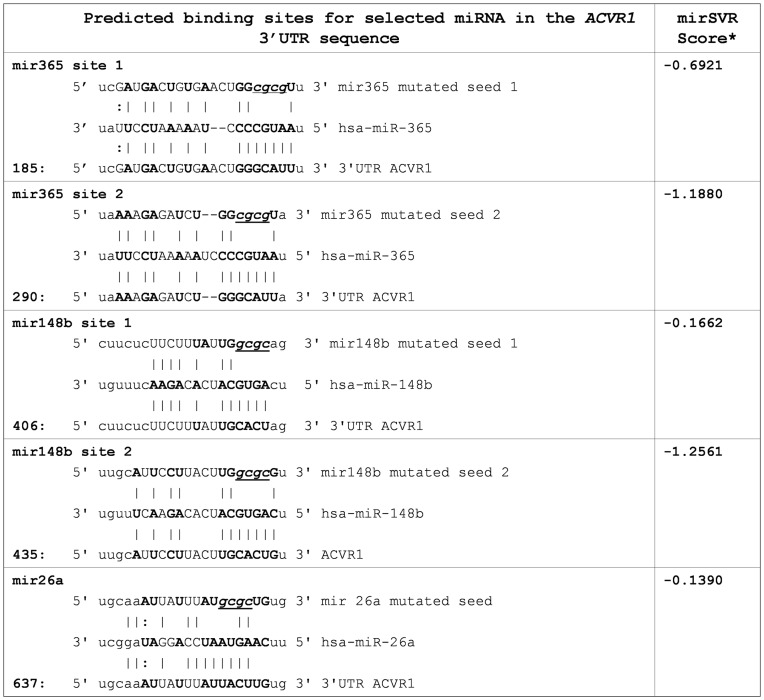
Predicted miR recognition sequences in the 3′UTR of the *ACVR1/Alk-2* **gene.** For each of the selected miRs, duplexes between miRNA sequences (middle) and wild-type (bottom) 3′UTR or harboring the corresponding mutated seed (top) are shown. Numbers on the left side indicate the matching starting position in the *ACVR1/Alk-2* 3′UTR, G:U wobble (**:**) and Watson-Crick pairing (**|**) are also indicated. miRVS scores rank the efficiency of the predicetd binding sites, good scores are ≤-0.1 [33].

To carry out experimental work we applied different criteria to choose candidate miRs: a) recognition of binding site by at least two different dedicated programs utilizing different selection methods; b) involvement in bone related processes. This allowed us to selected three miRs: hsa-miR-148b (mir148b), hsa-miR-365 (mir365) and hsa-miR-26a (mir26a) (Fig. S1 and Fig. 2). Binding sites for mir148b and mir365 were recognized by all the dedicated programs applied; mir26a, although predicted by the miRanda software only, was considered as an interesting candidate because of previous work indicating its involvement in the post-transcriptional regulation of the BMP signaling pathway during *in vitro* osteogenesis by targeting SMAD1 [7].

Considering the possibility of post-transcriptional regulation at the level of mRNA stability as predicted by *in silico* analysis, we have quantified the transcript by both semi-quantitative and quantitative RT-PCR (qRT-PCR) in C2C12 cells treated for different times with known transcription inhibitor Actinomycin D (ActD). As shown in Figure 1B and C, the *ACVR1/Alk-2* mRNA was significantly decreased after 3 and 6 hours in presence of the ActD transcription inhibitor. In particular, we found that expression of *ACVR1/Alk-2* was reduced by about 75 and 80% after 3 and 6 hours of treatment with ActD (Fig. 1C and Fig. S2, panel A, black diamonds). Conversely, the mRNA of the *ACVR1/Alk-2* gene appeared to be stable upon treatment of cells with the known translation inhibitor Cycloheximide (Chx), rather showing a slight but reproducible expression increase (Fig.S2, panel A, black squares). This phenomenon has been already described for genes known to have an unstable mRNA, and is considered due to the Chx effect on the synthesis of proteins involved in mRNA metabolism and stability [8], [9]. When cells were treated with both ActD and Chx in combination, we could still observe a time-dependent reduction in the *ACVR1/Alk-2* mRNA, although the slope of the decrease was attenuated (Fig. S2, panel A, grey triangles). Moreover, incubation of cells with Chx did not affect Luciferase activity by the expression construct carrying the 3′UTR sequence of the gene (Fig. S2, panel B).

**Figure 3:**
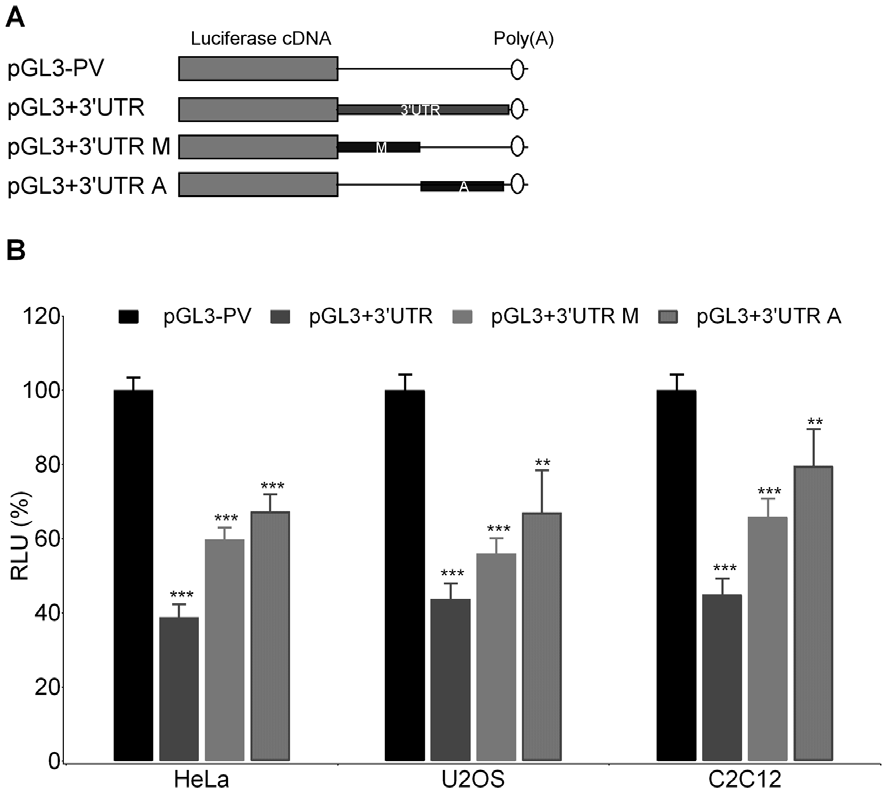
Effect of the presence of the *ACVR1/Alk-2* 3′UTR sequence on reporter gene expression. A) The whole *ACVR1/Alk-2* 3′UTR sequence and both the proximal and the distal derived fragments, carrying the miR binding sites containing module (3′UTR M) and the ARE sequences containing segment (3′UTR A), respectively, were subcloned into the pGL3 Promoter vector downstream the Luciferase coding sequence. B) After transient transfection in the indicated cell lines, reporter activity was measured and normalized against the pRL-SV40 expressing the Renilla Luciferase gene for transfection efficiency. Reporter gene activity of the construct carrying the *ACVR1-Alk2* 3′UTR (dark grey bars), or the M (pGL3+3′UTR M), and the A (pGL3+3′UTR A) fragments are reported as percentage of the activity obtained with the vector lacking the 3′UTR sequence (pGL3-PV, black bars) considered as 100%. Results are from at least two independent transfection experiments made in triplicate (RLU, Relative Luciferase Units). Two-tailed *Student*’*s t-test* was performed and significant differences in comparison to the activity of the pGL3-PV empty vector are given as: *p*<0.05*; *p*<0.01**; or *p*<0.001***.

To investigate the possible role of the 3′UTR region on post-transcriptional regulation of *ACVR1/Alk-2* expression, the 3′UTR sequence of *ACVR1/Alk-2* was subcloned into the pGL3-Promoter expression vector downstream of the Luciferase coding sequence (Fig. 3A). As indicated by our *in silico* analysis, the miR recognition sequences are mainly clustered in the proximal region of the *ACVR1/Alk-2* 3′UTR, therefore we also tested separately the functional contribution of the proximal half and of the distal region by generating two different Luciferase constructs (Fig. 3A, 3′UTR M and A). After transfection in different cell lines, the reporter gene activity was measured. As shown in Figure 3B, we found that the presence of the *ACVR1/Alk-2* 3′UTR sequence induced a decrease in Luciferase in all the tested cell lines, indicating that the region could affect the expression of the reporter gene at post-transcriptional level. Moreover, we found that both deletion fragments contribute to reduce the gene reporter expression, however, the strongest effect was observed with the whole 3′UTR sequence (Fig. 3B).

In order to verify if miRs play a role in the regulation of *ACVR1/Alk-2* expression by targeting its 3′UTR, as predicted by bioinfomatic means, the three selected miRs, mir148b, mir365 and mir26a commercially available as double stranded pre-miR (Table S2), were co-transfected with the pGL3-*ACVR1/Alk-2*-3′UTR construct in C2C12 and U2OS cell lines. The above miRs are expressed in these cell lines as well as in the HeLa cell line, although at different level (see Fig. S3).

**Figure 4:**
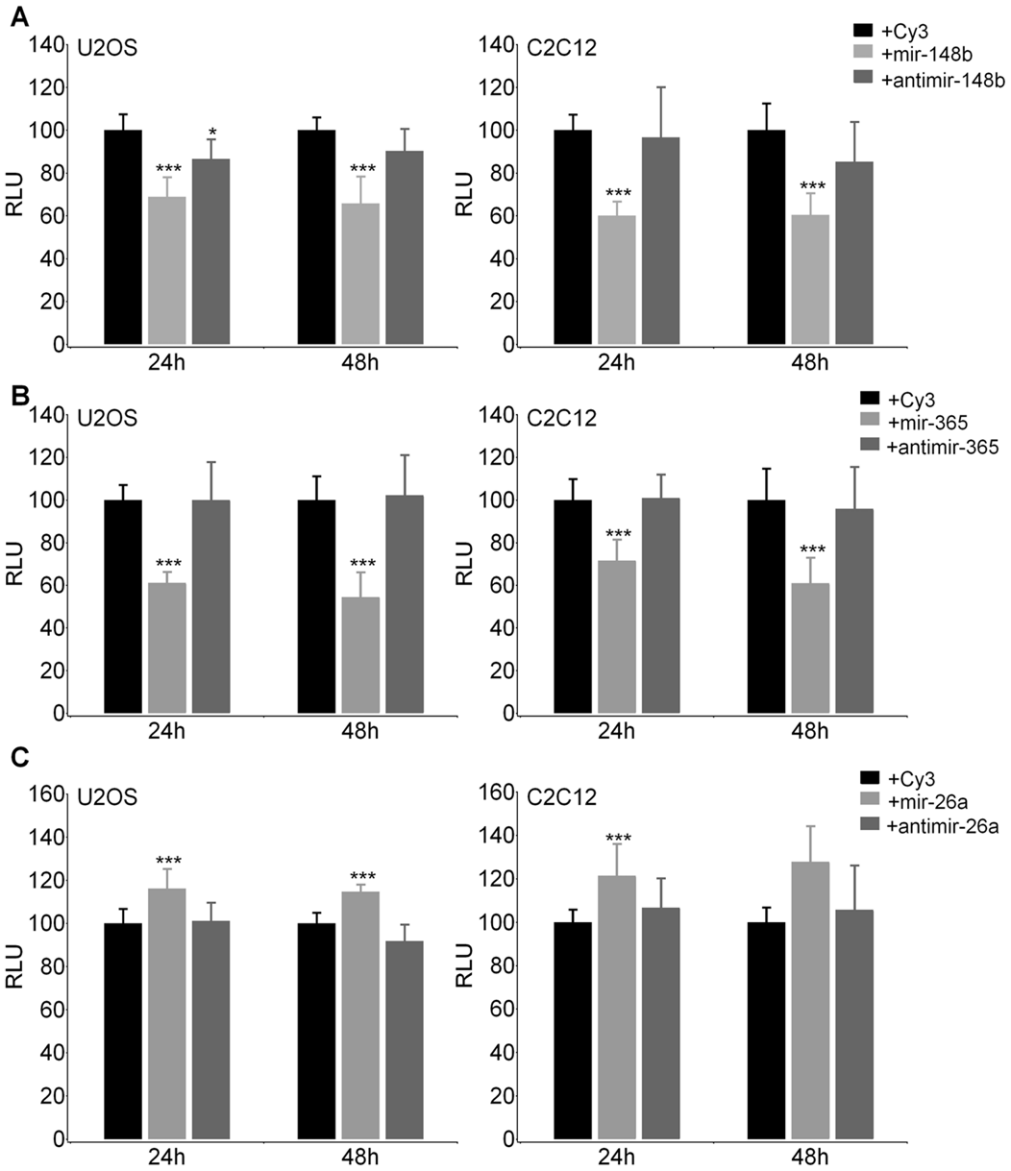
Effect of selected miRs on the expression of the *ACVR1/Alk-2* 3′UTR reporter construct. Relative Luciferase activity obtained after co-transfection of the *ACVR1/Alk-2* 3′UTR reporter construct with either a scrambled mir Cy3-conjugated as negative control (Black histograms), or each of the indicated miR (light grey), mir148b (A), mir365 (B) and mir26a (C) or with the corresponding anti-miR (dark grey histograms). The reporter gene activity measured in presence of the Cy3-miR negative control is considered as 100 for each independent experiment, RLU, Relative Luciferase Units. The statistical significance of the effect of miR compared to that obtained with the non targeting negative control was evaluated by *Student’s T-test* analysis, with *p*<0.05*; *p*<0.01**; or *p*<0.001***.

At 24 and 48 hours after transfection, the Luciferase activity was measured and compared to that obtained by transfecting cells with scrambled non-targeting pre-miR as negative control and the respective specific anti-miRs. As shown in Figure 4, transfection of mir148b (Fig. 4A) and mir365 (Fig. 4B) reduced the reporter gene activity, in both cell lines, compared to that detected after co-transfection of the pGL3-*ACVR1/Alk-2*-3′UTR construct with the scrambled miR used as negative control. The observed downregulation of the reporter gene activity was specific because was not observed with the control non-targeting miR and was reversed by the specific anti-miRs.

Conversely, we found that mir26a, co-transfected with pGL3-*ACVR1/Alk-2*-3′UTR construct, induced a significant increase in Luciferase activity compared to the control, suggesting a positive role on *ACVR1/Alk-2* expression (Fig. 4C).

The effect of miRs on Luciferase activity may be linked to an action on the stability of reporter gene mRNA mediated by the presence of the 3′UTR of *ACVR1/Alk-2* or, alternatively, to an effect on its translation. Therefore, we decided to evaluate the effect at the RNA level by studying the effect of the selected miR on the endogenous *ACVR1/Alk-2* mRNA by RT-qPCR.

**Figure 5:**
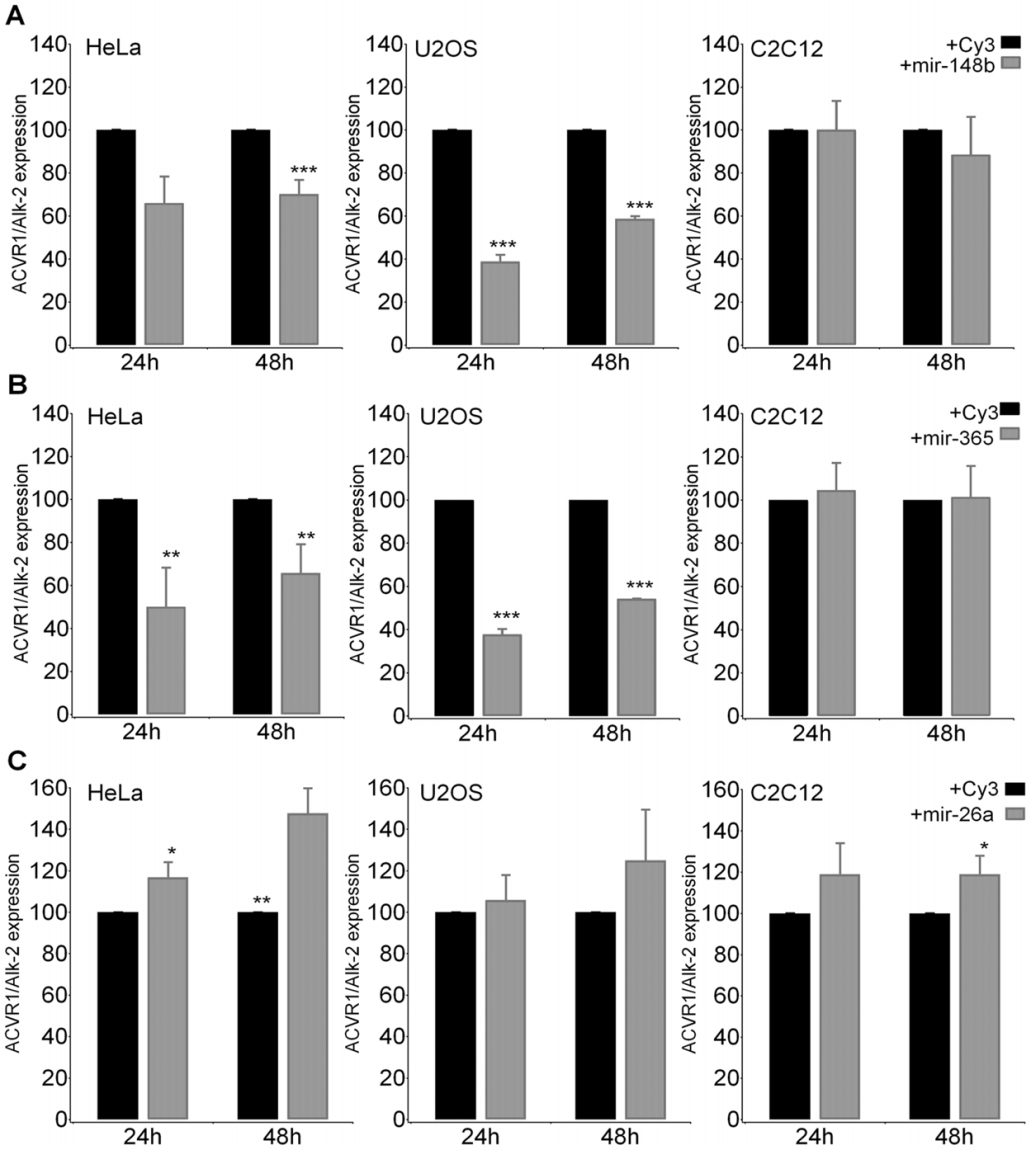
Effect of selected miRNAs on the expression of endogenous *ACVR1/Alk-2* mRNA. Selected miRs were transfected in the indicated cell lines and expression of *ACVR1/Alk-2* mRNA was measured by RT-qPCR and compared to the expression level of *ACVR1/Alk-2* transcript from cells transfected with a negative scrambled pre-miR control considered as 100. (**A**) miR148b; (**B**) miR365; (**C**) miR26a. Differences between the effect on *ACVR1/Alk-2* mRNA of specific miR compared to that of the Cy3-labeled negative control were evaluated by performing a *Student’s t-test* with *p*<0.05*, *p*<0.01**, *p*<0.001***.

As shown in Figure 5, we found that mir148b (Fig. 5A) and mir365 (Fig. 5B) induced a downregulation of *ACVR1/Alk-2* mRNA expression in U2OS and HeLa cells, both at 24 and 48 hours after transfection, with little if any effect in C2C12 cells. Conversely, and in accordance with reporter gene experiments, mir26a transfection caused an increase of *ACVR1/Alk-2* mRNA expression in the three tested cell lines (Fig. 5C).

**Figure 6:**
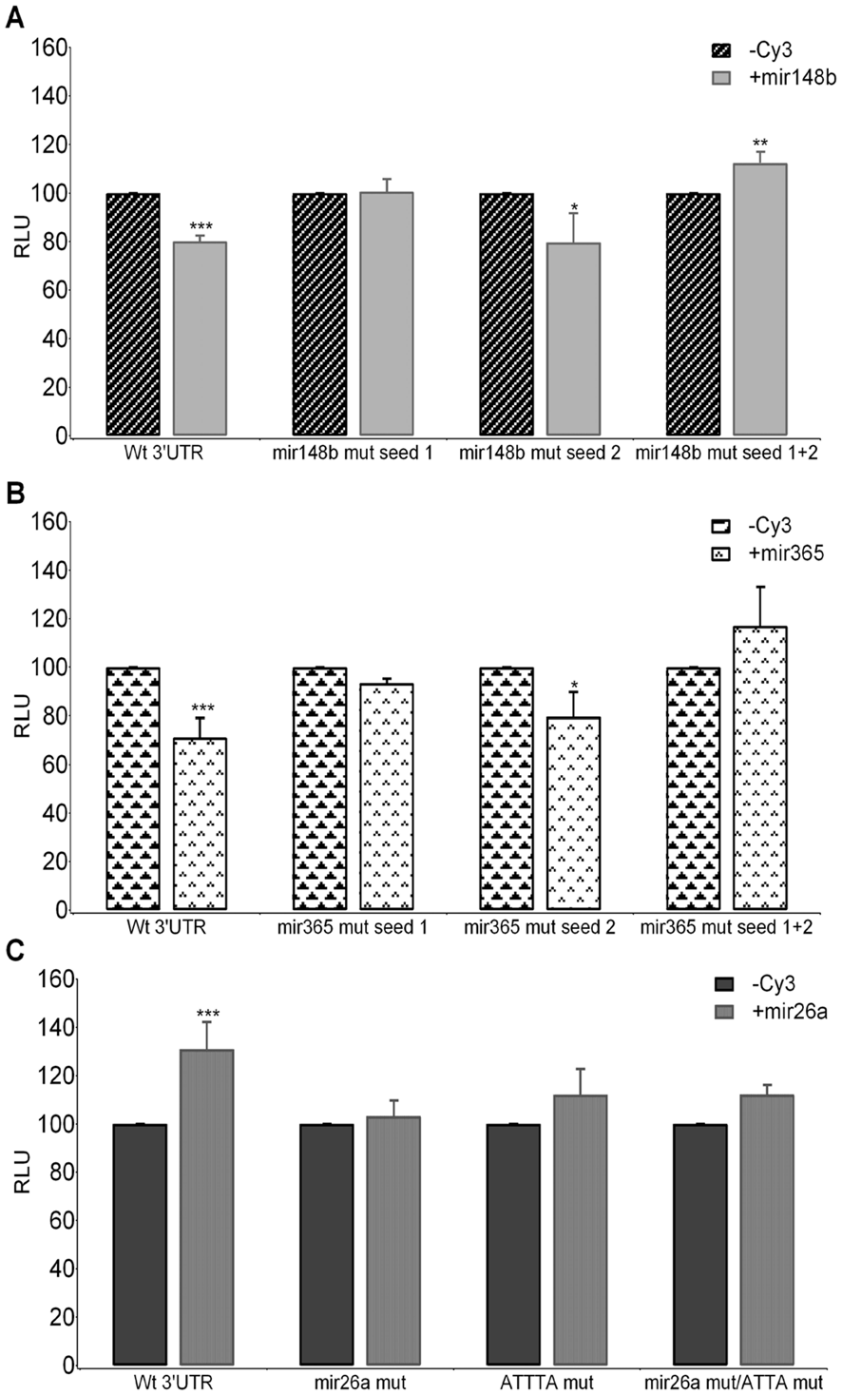
Binding site specific mutagenesis prevents the modulation of *ACVR1/Alk-2* expression operated by the selected miRs. Mutagenesis of miRs binding sites was introduced in the two seed sequences predicted for mir148b (A) and mir365 (B), indicated as Seed1 and Seed 2, and in the mir26a seed sequence (C). Site specific mutagenesis was also performed to abolish the first AUUUA element contained in the mir26a pairing region (ATTTA mut), alone or in combination with the mutation of the mir26a seed sequence (double mutant mir26 mut/ATTTA mut). Each pair of bars represents Luciferase activity in the presence of negative control miR (-), considered as 100%, or the specific miR (+). *Student’s t-test* was applied to evaluate differences between the effect of specific mir compared to that of the negative control for each construct, *p*<0.05*, *p*<0.01**, *p*<0.001***.

In order to further confirm the specificity of the effect of the selected miR on *ACVR1/Alk-2* expression, site directed mutagenesis was used to modify the miR target sites in the 3′UTR sequence by changing 4 nucleotides of the miR seed (Fig. 2). Each of the selected miRs was then transfected in combination with both mutated and wild-type pGL3-*ACVR1/Alk-2*-3′UTR constructs in U2OS cells, and the effect on Luciferase activity evaluated after 24 hours (Fig. 6). The three miRs under analysis exerted their own effect through the binding at the putative site recognized by predictive bioinformatic analysis. For mir148b two putative binding sites have been identified and subjected to mutagenesis. As shown in Fig. 6A, seed mutation of the first site prevented the action of mir148, that was on the contrary apparently unaffected by mutation of the second consensus site (see Fig. 2 for position and seed sequence).

Also for mir365 two different potential binding sites were predicted in the *ACVR1/Alk-2* 3′UTR (Fig. 6B). Mutagenesis of the single sites, in particular of the seed of the first recognition sequence, partially abolished the action of mir365. As observed for mir148b, when both mir365seeds were mutated we observed not only a rescue in Luciferase activity but also an increase in Luciferase activity, again suggesting that the two sites may cooperate and are necessary for mediating the action of the two miRs in targeting the 3′UTR of *ACVR1/Alk-2* gene.

Finally, seed mutation of the mir26a binding site prevented the positive effect on *ACVR1/Alk-2* expression mediated by the interaction of mir26a itself with its recognition sequence (Fig. 6C). As the pairing region of mir26a includes the first of the AUUUA modules (Au-rich element) mapping in the distal half of the 3′UTR, and it is reported that ARE sequences intervene and sometimes interfere with miRs in the post transcriptional regulation of gene expression, we have altered the corresponding ATTTA sequence by site directed mutagenesis and generated constructs carrying either the single ACCCA mutated element (ATTTA mut) or a double mutant carrying also the mir26a seed mutation (ATTTA mut/mir26a mut). After transfection with both the negative Cy3-labeled control and mir26a, Luciferase activity of the different constructs was evaluated. As shown in Figure 6C, change of the AUUUA motif both by itself or in combination with mutation of seed of mir26a, resulted in a partial loss of the positive effect observed in presence of mir26a, with no significant difference in the activity of the double mutant compared to that of the mir26a seed.

## Discussion

Regulation of gene expression is a crucial process in cell biology taking place by multiple control mechanisms at different levels. Recently, a growing body of the literature has focused on the role of miRs, as crucial players of post-transcriptional regulation of gene expression, both in physiological conditions and in disease [10]. miRs are mainly involved in negative regulation of gene expression through two basic modes of action, one based on mRNA decay and the other on translational repression (followed by mRNA decay). A complicating factor is that these processes are closely linked in the cell [1].

Mechanisms and factors intervening in the regulation of *ACVR1/Alk-2* gene expression are still poorly investigated, although the encoded protein, a type I receptor for BMPs, is involved in fundamental biological processes during embryo development, osteogenic differentiation of progenitor mesenchymal cells [11]–[15], and various pathological conditions including cancer [16], [17]. Since mutations in the gene, proved to induce gain of function, are associated with the severely disabling FOP for which no specific treatment is available, understanding mechanisms of regulation should increase the identification of possible targets for new therapeutic strategies.

The pathogenic mechanisms responsible for FOP are still largely unclear. Moreover, the histological features of the early FOP lesions and the observation that stimuli such as trauma or infections may initiate or exacerbate the acute phases of the disease [18], suggests that inflammation and the immune response, together with the presence of the mutated receptor, are a central issue in FOP.

In this work we focused our attention on the role of the 3′UTR sequence in the modulation of the *ACVR1/Alk-2* gene expression at post-transcriptional level. We found that the introduction of the 3′UTR region of *ACVR1/Alk-2* in a Luciferase reporter gene assay was able to induce a decrease of its expression, supporting the idea that the region could be involved in the regulation of gene expression.

Data present in GenBank indicate that *ACVR1/Alk-2* transcript is expressed in many different cell types raising the question whether many different cell types might possess different mechanisms to modulate its expression according to the biological processes in which they are involved.

Our bioinformatic analysis on the 1.1 kb 3′UTR region suggested the presence of sequences or conserved structural motifs with a potential functional role in post-transcriptional regulation of *ACVR1/Alk-2* expression. We found that the 3′UTR region may be divided in two putative functional modules characterized by a proximal half containing most of the predicted binding sites for miRs and a more distal sequence harboring several AUUUA pentamers.

Our finding that *ACVR1/Alk-2* mRNA is unstable in presence of transcription inhibitors supported the bioinformatic prediction and our hypothesis that it could be subjected to post-transcriptional regulation mechanisms, and, at this level, one of the possible mechanisms involves the action of miRs. Moreover, a recently published article reported that *ACVR1/Alk-2* is repressed by binding of mir30c to its 3′UTR, during adipocytes differentiation [19].

Selection of miRs among those suggested by *in silico* analysis was based on prediction by different specialized programs for mir148b and mir365 (see Materials and Methods section) at two different and conserved putative binding sites. For mir26a, binding was predicted only by one program, the miRanda software, however, as already mentioned we considered it an interesting candidate [7].

For mir148b and mir365 our findings were in accordance with the effect of most miRs, since they were able to downregulate *ACVR1/Alk-2* expression, whereas mir26a exerted an unexpected positive effect. All three miRs acted by binding the predicted consensus sequences, as demonstrated by the abolition of their effects after mutagenesis of the respective binding elements.

mir148b belongs to the mir148 family of miRs comprising also mir148a and mir152 (see [20]). They largely share the consensus binding site sequence, in particular seeds of mir148a and 148b differ by two nucleotides in position 7. We decided to focus on mir148b since it was reported to exert an effect on: osteogenic differentiation of mesenchimal stem cells [21]; tumorigenic processes [22], [23] and the innate immune response [20].

Studies on mir365 indicate that it can be involved in regulation of genes involved in the control of cell cycle and senescence [24]; in apoptosis of endothelial cells [25]; in brown fat differentiation by inhibiting myogenesis [26]; in triggering chondrocytes proliferation and maturation [27]; in targeting the expression of IL-6 gene [28].

Regarding mir 26a, it was reported to affect the osteogenic differentiation of human adipose tissue-derived stem cells induced by BMP treatment. Its expression increases progressively during the differentiation process and, in terminal differentiated cells, contributes to silencing of the BMP pathway by targeting the SMAD1 protein [7]. It is considered a tumor suppressor gene as its expression is downregulated in different form of cancer and this correlates with poor prognosis [29]–[31]. In the immune system, mir26a appears to intervene in the IFNβ pathway in primary macrophages [32].

mir26a showed an upregulating effect on *ACVR1/Alk-2* expression, contrary to the most typical effect of miRs, mainly inducing negative regulation of gene expression. A similar “paradoxical” upregulating effect of miR was described in condition of response to particular stimuli, such as culture in serum deprivation, for the TNFα gene, which is highly regulated at post-transcriptional level, by mechanisms correlated with the presence of particular sequence elements rich in A and U nucleotides (AU-rich Elements, ARE) in the 3′UTR region [33], [34].

The *ACVR1/Alk-2* 3′UTR sequence has a structure highly reminiscent of that of the TNFα gene and harbors five AUUUA motifs, the first of which is located at the mir26a pairing region, immediately upstream of the seed (see Fig. S1). We propose that the apparent positive regulation of *ACVR1/Alk-2* induced by mir26a could be due to interaction of these two functional elements (mir26a binding site and ARE). Results of mutational analyses indicated that mutagenesis of the AUUUA motif, induced a decrease of the effect of mir26a on Luciferase activity both by itself and in combination with the mutation of the mir26a seed, probably as a result of the perturbation of the pairing region for mir26a.

As the role of inflammation and of the immune system is relevant for FOP pathogenesis, it is very interesting to note that the structural and functional features of the 3′UTR sequence of *ACVR1/Alk-2*, are reminiscent of what is observed for genes encoding cytokines or molecules involved in inflammation pathways, thus suggesting for *ACVR1/Alk-2* expression similar mechanisms of regulation. Indeed, many cytokine mRNAs are expressed as short lived transcripts subjected to highly regulated post-transcriptional controls, involving the presence of miR binding sites and ARE motifs that can affect mRNA stability [35].

Our work highlights the relevance of miRs in gene expression regulation, and in particular, we provide the first evidence that mir148b, mir365 and mir26a can target *ACVR1/Alk-2* expression. miRs are considered valuable cellular targets for the development of specific and innovative therapeutic interventions and this could be of particular interest for FOP, as no specific treatment is presently available to cure the disease.

## Materials and Methods

### Bioinformatic Analysis

Extensive bioinformatic analysis of *ACVR1/Alk-2* 3′UTR sequence was performed using different programs available on line. The conservation profile was obtained with the tools for comparative genomics of the VISTA genome Browser (http://pipeline.lbl.gov/cgi-bin/gateway).

Four different programs were applied to identify putative sites for miRs: TargetScan5.1 (http://www.targetscan.org/), PicTar (http://pictar.mdc-berlin.de/) miRBase (http://www.mirbase.org/), and miRanda (http://www.microrna.org). For each miR we have indicated the mirSVR score that ranks the efficiency of miRanda predicted binding sites (Fig. 2) [36]. We also used a program for AU-rich elements-containing sequence detection available at http://brp.kfshrc.edu.sa/ARED/.

### PCR Amplification of ACVR/Alk-2 3′UTR Sequence

The 3′UTR region of the *ACVR1/Alk-2* gene (UCSC Feb 2009, chr2∶158,592,958-158,594,042) was obtained by PCR from genomic DNA of a control individual with the oligonucleotides specified in Table S1. The products corresponding to the entire 3′UTR sequence or to the derived fragments (M and A, see Fig. 3A) were inserted in the XbaI restriction site of the pGL3 Promoter Vector (Promega), downstream of the Luciferase reporter cDNA. Constructs were checked by sequencing.

### Cell Culture, Treatment and Transfection

Different established cell lines were used for experiments: HeLa, U2OS, and C2C12 cells. These cell lines were already available in our laboratory and were originally purchased from ATCC (ATCC-LGC Standards Partnership). Cells were cultured in DMEM medium with 10% FBS, penicillin-streptomycin (50 U/ml) and 2 mM glutamine. HeLa cells were cultured in MEM medium with 10% FBS, penicillin-streptomycin (50 U/ml), 2 mM glutamine, 1 mM sodium pyruvate and 1 mM non essential aminoacids. Cells were grown at 37°C with 5% CO_2_.

To check the stability of the *ACVR1/Alk-2* mRNA, C2C12 cells, were treated for different times (0–3–6 hours) in presence or absence of transcription inhibitors Actinomycin D 10 µg/ml (ActD, Sigma) and Doxorubicin 1 µM (Dox, Sigma) (not shown), and the mRNA expression was quantified by quantitative RT-qPCR. C2C12 cells were also treated with the protein synthesis inhibitor Cycloheximide at 10 µg/ml (Chx, Enzo Life Sciences) both as single treatment or in combination with ActD.

To carry out the functional study, reporter constructs carrying the entire 3′UTR of *ACVR1/Alk-2* and the two derived fragments, were transfected in the indicated cell lines with the Lipofectamine 2000 transfecting agent according to the protocol provided by the manufacturer (Invitrogen, Life Technology). In order to normalize the transfection efficiency, the reporter constructs under analysis were always transfected together with the pRL-SV40 Renilla expression vector. Each experiment was performed in triplicate.

Subsequently, to investigate the possible role of miR on post-transcriptional regulation of *ACVR1/Alk-2* expression, we selected three miRs by bioinformatic analysis, filtered on the basis of the conservation of their binding sites among species and recognized by at least two of the softwares used.

Selected miRs were transfected in the different cell lines as commercially available double stranded pre-miR molecules (pre-MIR, Ambion, Life Technology), cells were also transfected with a negative control (AM17120 Cy3 labeled pre-miR negative control n. 1, Ambion, Life Technology,) or with the corresponding anti-miR molecule (Ambion, Life Technology) (Table S2).

After 24 and 48 hours post-transfection, cells were harvested and processed for assessing the Luciferase activity or for total RNA extraction to be used in RT-qPCR experiments.

The Dual Luciferase Reporter Assay System from Promega was used to measure Luciferase activity according to the provided protocol. For Luciferase assay a minimum of two independent transfection experiments were performed, with each point in triplicate.

### RNA Extraction and cDNA Synthesis

Total RNA was extracted from cells using the RNeasy Mini Kit from Qiagen. RNA quantity was measured with Nanodrop Spectrophotometer (Thermoscientific), and first strand cDNA was synthesized with the Advantage RT-for-PCR Kit (Becton Dickinson) according to the manufacturer’s instructions.

### Quantitative Reverse Transcription PCR (RT-qPCR)

The effect of individual miRs on endogenous *ACVR1/Alk-2* transcript was evaluated through RT-qPCR using specific TaqMan Gene Expression Assay (Applied Biosystems) (Table S2). Samples were measured in triplicate and the results were normalized using TaqMan Gene Expression Assays for two reference genes: β-Actin and GAPDH. qPCR was run on the IQ5 instrument from BioRad, and Data analysis was performed using the provided Bio-Rad iQ5 software for Gene Expression Study.

### Mutagenesis

Quikchange Lightning site-directed mutagenesis kit from Stratagene was used to mutagenize four bases in the seed of each of the the miR binding sites in *ACVR1-Alk2* 3′UTR constructs (as indicated in Fig. 2), following the provided protocol. Oligonucleotides used in mutagenesis are indicated in Table S1.

### miRNA Endogenous Expression

The RNA fraction containing miRNA was isolated from the different cell lines HeLa, C2C12, and U2OS, by using mirVana miRNA Detection kit (Ambion), cDNA was synthesized with TaqMan MicroRNA Reverse Transcription Kit and the expression profile of mir148b, mir365 and mir26a was evaluated by RT-qPCR (Table S2 for probe specification), using specific TaqMan MicroRNA Assays (Applied Biosystems). Each assay was performed in triplicate.

### Statistical Analysis

All the experiments were performed in triplicate and repeated at least twice independently (two - up to five times). Where indicated Unpaired *Student’s* t*-Test* (two tails) was applied to verify statistical significance of the observed variations.

## Supporting Information

Figure S1 **Comparison between Human and mouse** ***ACVR1/Alk-2*** **3**′**UTR sequences (Clustal alignment).** * indicates the full conservation of the corresponding nucleotide position. Pairing region of selected miRs and the ARE sequences-containing module are highlighted (light grey and dark grey respectively).
(DOC)

Figure S2 **Effect of inhibition of protein synthesis on** ***ACVR1/Alk-2*** **expression.** A) *ACVR1/Alk-2* mRNA expression level in C2C12 cells cultured as follows: basal conditions (untreated, UN, open squares), treated with 10 µg/ml Cycloheximide (Chx) for the indicated time points (Chx, black squares); with 10 µg/ml Actinomycin (ActD, black diamonds) or treated with both ActD and Chx in combination (ActD/Chx, grey triangles). B) Luciferase Activity measured in C2C12 cells transfected with the pGL3-3′UTR expression construct and then treated with Chx as indicated in A. UN, untreated cells, RLU, Relative Luciferase Units.
(TIF)

Figure S3 **Expression leves of selected miRs in the cell lines used in transfection experiments.** A) The expression profile of mir148b, mir365 and mir26a was determined by RT-qPCR in the different cell lines used. Average expression levels were normalized to snRNA RNU44 and Z30, for human cells (HeLa and U2OS), and to snoRNA202 for mouse cells C2C12. B) Table reporting the mean Ct values and the corresponding standard deviation (SD) for each miR and in the indicated cell lines.
(TIF)

Table S1 **List of oligonucleotides used in this work.**
(DOC)

Table S2 **List and ID specification of TaqMan assays used for RT-qPCR experiments and of pre-miR and anti-miR molecules used in transfection experiments.** Sequences of mature miRs are also indicated.
(DOC)
