# Supplementary material for: The Role of the 3′UTR Region in the Regulation of the ACVR1/Alk-2 Gene Expression
Source: PLoS One. 2012 Dec 5;7(12):e50958. doi: 10.1371/journal.pone.0050958 (PMC3515447; doi:10.1371/journal.pone.0050958)
Supplement: Figure S1 — Comparison between Human and mouse ACVR1/Alk-2 3′UTR sequences (Clustal alignment). * indicates the full conservation of the corresponding nucleotide position. Pairing region of selected miRs and the ARE sequences-containing module are highlighted (light grey and dark grey respectively). (DOC) [file pone.0050958.s001.doc]

**Figure S1**

h3'UTR CATTTTCAT-AGTGTCAAGAAGGAAGATTTGACGTTGTTGTCATTGTCCAGCTGGGACCT 59

m3'UTR CCTTGTCACCGGTGTCAAGAAGGAGAGT----CAATGCTGTCCTTGTCCAGCTGGGACCT 56

* ** *** ************* * * ** **** *****************

h3'UTR AATGCTGGCCTGACTGGTTGTCAGAATGGAATCCATCTGTCTCCCTCCCCAAA-TGGCTG 118

m3'UTR AATGCTGGCCTGACTGGTTGTCAGAACAGAATCCATCTGACCCCCTTCCCGAAGTGGCTG 116

************************** *********** * **** *** ** ******

h3'UTR CTTTGACA-AGGCAGACGTCG-TACCCAGCCATGTGTT--GGGGAGACATCAAAACCACC 174

m3'UTR CTTTGACGGAAGCAGATGTCTCTTCCCAGCCATGTTCCAGGGGGAGACACCAAAACCACC 176

******* * ***** *** * *********** ********* **********

mir365 site 1

h3'UTR CTAACCTCGCTCGATGACTGTGAACT***GGGCATT***TCACGAACTGTTCACACTGCAGAGACT 234

m3'UTR CTAACCTCGCTCAAAAACTGTGACTC***G***A***GC***CC***T***CGATGAACTGTTCACACCACAAAGACT 236

************ * ******* * ** * * ************* ** *****

h3'UTR -AATGTTGGACAGACACTGTTGCAAAGGTAGGG--ACTGGAGGAACACAGAGAAATCCTA 291

m3'UTR TAACGGTGGGCAGGTCTGGTGGCAAGGGGGAGGGAAGTGGAGGAACCCGGAAAGATCCTG 296

** * *** *** ** **** ** ** * ********* * ** * *****

mir365 site 2

h3'UTR AAAGAGATCT***GGGCATT***AAGTCAGTGGCTTT--GCATAGCTTTCACAAGTCTCCTAGACA 349

m3'UTR CAGGCGATCT***GGGCATT***AAGACAGTGGCTCTCTGCGTATCTTTCGCGGGTCTCCTAGACA 356

* * *************** ******** * ** ** ***** * ************

mir148b site 1

h3'UTR CTCCCCACGGGAAACTCAAGGAGGTGGTGAATTTTTAATCAGCAATATTGCCTGTGCTTC 409

m3'UTR CTCCCCACGGGAAGCTCAAGGAGGCGGTGAATTCGTAATCAGCAATATCGGCT--GCATC 414

************* ********** ******** ************* * ** ** **

mir148b site 1 mir148b site 2

h3'UTR TCTTCTTTAT***TGCACT***AGGAATTCTTTGCATTCCTTACT***TGCACTG***TTACTCTTAATTTT 469

m3'UTR TACTCTTCGT***TGCACT***AGGAATTCTGTGCATTCCTTACT***TGCACTG***TGGCCCTTAATCTT 474

* **** **************** ********************* * ****** **

h3'UTR AAAGACCCAACTTGCCAAAATGTTGGCTGCGTACTCCACTGGTCTGTCTTTGGATAATAG 529

m3'UTR AAAGACCCAACTTGCCAAAACATTGGCTGCGTACTCCACTGGCCTGTCTCTGGATAATAG 534

******************** ******************** ****** **********

h3'UTR GAATTCAATTTGGCAAAACAAAA-TGTAATGTCAGACTTTGCTGCATTTTACACATGTGC 588

m3'UTR GAATTCAATCTGGCAACACAAAAATGTACCGTTGGACTCTGCTGCATTTTACACACGTGC 594

********* ****** ****** **** ** **** **************** ****

mir26a

h3'UTR TGATGTTTACAATGATGCCGAACATTAGGAATTGTTTATACACAACTTTGCAAATTATTT 648

m3'UTR TGATGTTTACAAGGATGC-GAACATTAGGAATTGTTTAGACACAACTTTGCAAATTATTT 653

************ ***** ******************* *********************

h3'UTR ***ATTACTTG***TGCACTTAGTAGTTTTTACAAAACTGCTTTGTGCATATGTTAAAGCTTATTT 708

m3'UTR ***ATTACT***G***G***TGCACTTAGCGGTTTGTTTGAAACCGCCTCGTGCATATGTTAAAGCTTATTT 713

****** ********** **** * **** ** * **********************

h3'UTR TTATGTGGTCTTATGATTTTATTACAGAAATGTTTTTAACACTATACTCTAAAATGGACA 768

m3'UTR TTATGTGGTCTTATGATTTTATTACCGAAATGTTTTTAACACCCAACTCTGAAACGGACA 773

************************* **************** ***** *** *****

h3'UTR TTTTCTTTTATTATCAGTTAAAATCACATTTTAAGTGCTTCACATTTGT--------ATG 820

m3'UTR TTTTCTTTTATTATCAGTTAAATTCACATTT-AAGTGCTTCACATTTTTTTTTTTAAATG 832

********************** ******** *************** * ***

h3'UTR TGTGTAGACTGTAACTTT-TTTTCAGTTCATATGCAGAACGTATTTAGCCATTACCCACG 879

m3'UTR TGTGTAGACTGTAACTTTCTTTTCAGTTCGTATGCAGAACATATTTAGCCATTACCCATG 892

****************** ********** ********** ***************** *

h3'UTR TGACACCACCGAATATATTACTGATTTAGAAGCAAAGATTTCAGTAGAATTTTAGTCCTG 939

m3'UTR CAACACCACCCGATATATTACTGATTTAGAAGCAAAGATTTCAGTAGAATTTTAGTCCCA 952

******** **********************************************

h3'UTR AACGCTACGGGGA-AAATGCATTTTCTTCAGAATTATCCATTACGTGCATTTAAACTCTG 998

m3'UTR AACGCTGTGGGGGGAAATGCATCTTCTTCGGAATTATCCATTACGTGCATTTAAACTCTG 1012

****** **** ******** ****** ******************************

h3'UTR CCAGAAAAAAA--TAACTATTTTGTTTTAATCTACTTTTTGTATTTAGTAGTTATTTGTA 1056

m3'UTR CCAGAAAAAAAAATAACTATTTTGTTTTAATCTACTTTTTGTATTTAGTAGTTATTTGTA 1072

*********** ***********************************************

h3'UTR TAAATTAAATAAACTGTTTTCAAGTCAAAAAAAAAAAAAAAAAAAA 1102

m3'UTR TAAATTAAATAAACTGTTTTCAAGTCAAAAAAAAAAAAAAAAA--- 1115

*******************************************
