# Supplementary figures and images for: The Role of the 3′UTR Region in the Regulation of the ACVR1/Alk-2 Gene Expression
Source: PLoS One. 2012 Dec 5;7(12):e50958. doi: 10.1371/journal.pone.0050958 (PMC3515447; doi:10.1371/journal.pone.0050958)

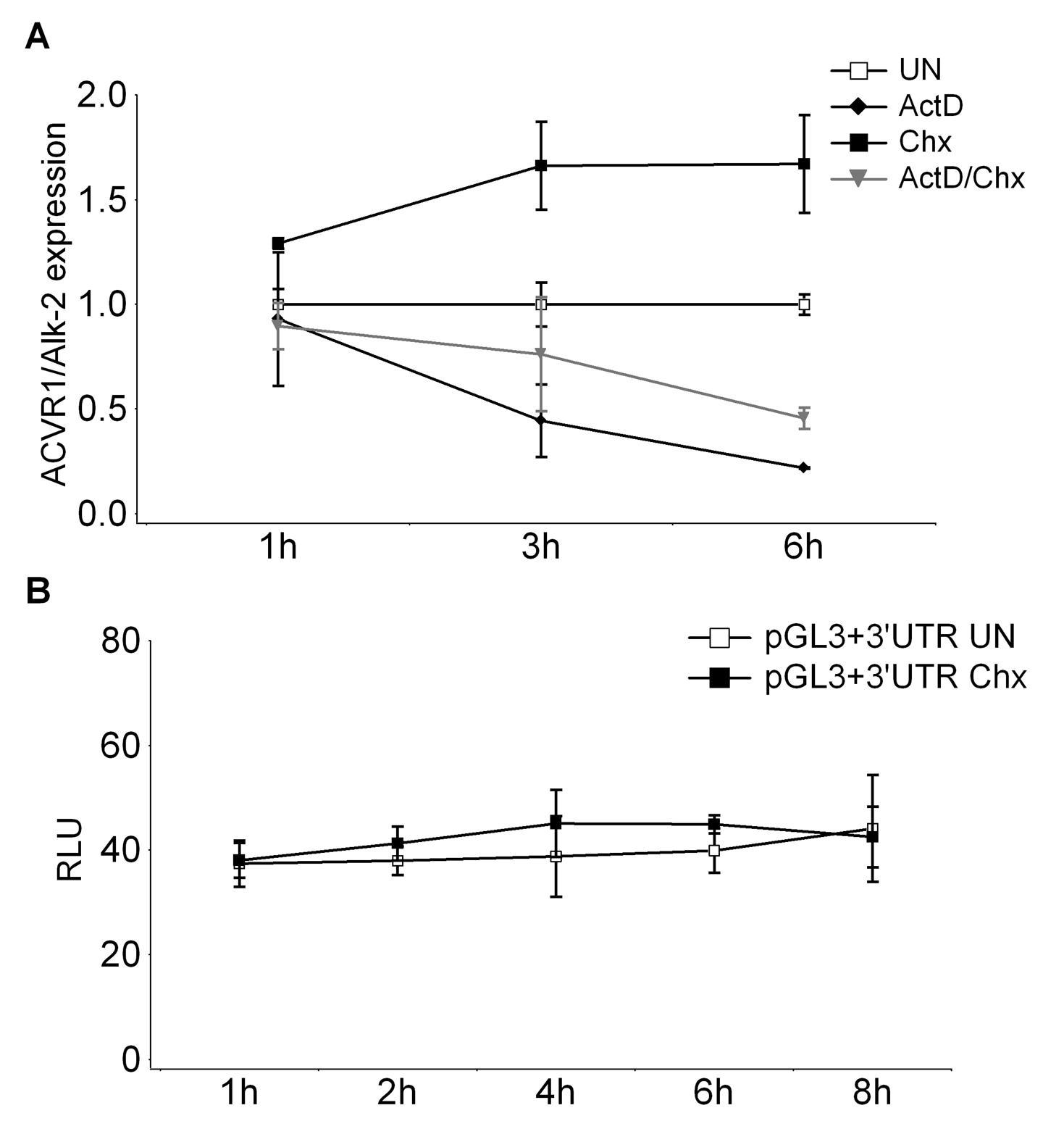

Supplement: Figure S2 — Effect of inhibition of protein synthesis on ACVR1/Alk-2 expression. A) ACVR1/Alk-2 mRNA expression level in C2C12 cells cultured as follows: basal conditions (untreated, UN, open squares), treated with 10 µg/ml Cycloheximide (Chx) for the indicated time points (Chx, black squares); with 10 µg/ml Actinomycin (ActD, black diamonds) or treated with both ActD and Chx in combination (ActD/Chx, grey triangles). B) Luciferase Activity measured in C2C12 cells transfected with the pGL3-3′UTR expression construct and then treated with Chx as indicated in A. UN, untreated cells, RLU, Relative Luciferase Units. (TIF) [file pone.0050958.s002.tif]

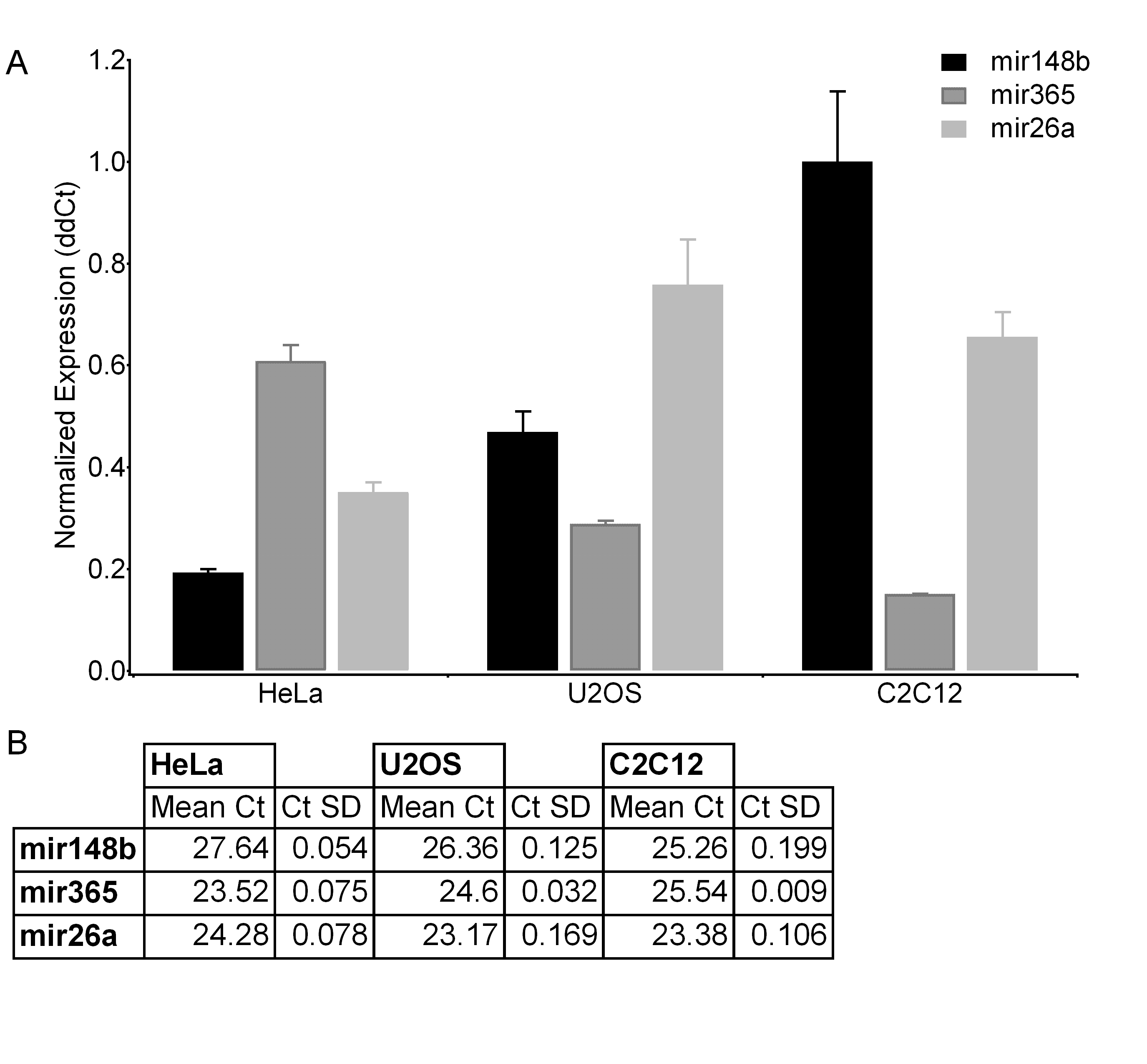

Supplement: Figure S3 — Expression leves of selected miRs in the cell lines used in transfection experiments. A) The expression profile of mir148b, mir365 and mir26a was determined by RT-qPCR in the different cell lines used. Average expression levels were normalized to snRNA RNU44 and Z30, for human cells (HeLa and U2OS), and to snoRNA202 for mouse cells C2C12. B) Table reporting the mean Ct values and the corresponding standard deviation (SD) for each miR and in the indicated cell lines. (TIF) [file pone.0050958.s003.tif]
