# Supplementary material for: The Role of the 3′UTR Region in the Regulation of the ACVR1/Alk-2 Gene Expression
Source: PLoS One. 2012 Dec 5;7(12):e50958. doi: 10.1371/journal.pone.0050958 (PMC3515447; doi:10.1371/journal.pone.0050958)
Supplement: Table S1 — List of oligonucleotides used in this work. (DOC) [file pone.0050958.s004.doc]

Table S1

| **Primer name** | **Sequence of Oligonucleotides used in this work** |
| --- | --- |
| 3'UTR-XBA-F | TCTAGACCCTCGACAAATTGAAAACTGAC |
| 3'UTR-XBA-R | TCTAGACTGGCAGAGTTTAAATGCACG |
| ARE-XBA-1F | TCTAGAGAATTGTTTATACACAACTTTGC |
| ARE-XBA-2R | TCTAGAGACTTGAAAACAGTTTATTTAATTT |
| mir-148b F | CTAGGAATTCTTTGCATTCCTTACTTGGCGCGTTACTCTTAATTTTAAAGACCCAAC |
| mir-148b R | GTTGGGTCTTTAAAATTAAGAGTAACGCGCCAAGTAAGGAATGCAAAGAATTCCTAG |
| mir-148b 2F | GCCTGTGCTTCTCTTCTTTATTGGCGCAGGAATTCTTTGCATTCC |
| mir-148b 2R | GGAATGCAAAGAATTCCTGCGCCAATAAAGAAGAGAAGCACAGGC |
| mir-26a F | CACAACTTTGCAAATTATTTATGCGCTGTGCACTTAGTAGTTTTTACAAAACTGC |
| mir-26a R: | GCAGTTTTGTAAAAACTACTAAGTGCACAGCGCATAAATAATTTGCAAAGTTGTG |
| mir-365 F1 | GTCCGATGACTGTGAACTGGCGCGTTCACGAACTGTTCAC |
| mir-365 R1 | GTGAACAGTTCGTGAACGCGCCAGTTCACAGTCATCGAGC |
| mir-365 F2 | TCCTAAAAGAGATCTGGCGCGTAAGTCAGTGGCTTTGCATAGC |
| mir-365 R2 | GCTATGCAAAGCCACTGACTTACGCGCCAGATCTCTTTTAGGA |
