# Supplementary material for: The Role of the 3′UTR Region in the Regulation of the ACVR1/Alk-2 Gene Expression
Source: PLoS One. 2012 Dec 5;7(12):e50958. doi: 10.1371/journal.pone.0050958 (PMC3515447; doi:10.1371/journal.pone.0050958)
Supplement: Table S2 — List and ID specification of TaqMan assays used for RT-qPCR experiments and of pre-miR and anti-miR molecules used in transfection experiments. Sequences of mature miRs are also indicated. (DOC) [file pone.0050958.s005.doc]

**Table S2**

| **Gene** | **TaqMan Gene Expression Assay (Species and ID specification)** | | | |
| --- | --- | --- | --- | --- |
| *ACVR1/Alk2* | *Homo sapiens* | | Hs00153836_m1 | |
| *acvr1/alk2* | *Mus musculus* | | Mm00431645_m1 | |
| *Beta-Actin* | *Homo sapiens* | | Hs99999903_m1 | |
| *Beta-Actin* | *Mus musculus* | | Mm00607939_1 | |
| *GADPH* | *Homo sapiens* | | Hs99999905_m1 | |
| *gadph* | *Mus musculus* | | Mm99999915_g1 | |
| snRNA RNU44 | *Homo sapiens* | | 001094 | |
| Z30 | *Homo sapiens* | | 001092 | |
| snoRNA202 | *Mus musculus* | | 001242 | |
| mir148b | *Homo sapiens/Mus musculus* | | 000471 | |
| mir365 | *Homo sapiens/Mus musculus* | | 001020 | |
| mir26a | *Homo sapiens Homo sapiens/Mus musculus* | | 000405 | |
| **Micro RNA** | **MiRBase Mature miRNA Accession ID** | **Mature miRNA Sequence** | **Ambion Product ID Pre-MIR** | **Ambion Product ID anti-MIR** |
| hsa-miR-148b | MIMAT0000759 | UCAGUGCAUCACAGAACUUUGU | PM 10264 | [AM10264](https://products.appliedbiosystems.com:443/ab/en/US/adirect/ab?cmd=ABAssayDetailDisplay&assayID=AM10264&Fs=y&adv_phrase3=EXACT&miRNAType=antimir&adv_phrase2=EXACT&adv_phrase1=EXACT&assayType=apmiRNA&catID=603009&adv_kw_filter3=ALL&srchType=keyword&adv_kw_filter2=ALL&SearchRequest.Common.QueryText=hsa-mir-148b&adv_kw_filter1=ALL&adv_query_text3=&searchType=keyword&adv_query_text2=&adv_query_text1=&uploadType=ID+List&adv_boolean3=AND&adv_boolean2=AND&adv_boolean1=AND&chkBatchQueryText=false&kwfilter=ALL&SearchRequest.Common.PageNumber=1&msgType=ABAntiPremiRNAKeywordResults) |
| hsa-miR-365 | MIMAT0000710 | UAAUGCCCCUAAAAAUCCUUAU | PM 11133 | AM11133 |
| hsa-miR-26a | MIMAT0000082 | UUCAAGUAAUCCAGGAUAGGCU | PM 10249 | AM10249 |
